# Owning the body in the mirror: The effect of visual perspective and mirror view on the full-body illusion

**DOI:** 10.1038/srep18345

**Published:** 2015-12-17

**Authors:** Catherine Preston, Benjamin J. Kuper-Smith, H. Henrik Ehrsson

**Affiliations:** 1 Psychology Department, University of York, York, YO10 5DD, UK; 2 Brain, Body and Self Laboratory, Department of Neuroscience, Karolinska Institutet, Stockholm, Sweden

## Abstract

Mirrors allow us to view our own body from a third-person (observer) perspective. However, how viewing ourselves through a mirror affects central body representations compared with true third-person perspective is not fully understood. Across a series of experiments, multisensory full-body illusions were used to modulate feelings of ownership over a mannequin body that was viewed from a third-person perspective through a mirror, from a third-person perspective without a mirror, and from a first-person perspective. In contrast to non-mirror third-person perspective, synchronously touching the participant’s actual body and the mannequin body viewed in the mirror elicited strong feelings of ownership over the mannequin and increased physiological responses to the mannequin being threatened compared to the equivalent asynchronous (non-ownership) control condition. Subjective reports of ownership viewing the mannequin through a mirror were also statistically equivalent to those following the first-person perspective illusion. These findings suggest that mirrors have a special role for viewing the self. The results also support the importance of egocentric reference frames for body ownership and suggest that mirror reflections of one’s own body are related to peripersonal space, which enables updating of central body representations.

Multisensory body illusions enable experimental investigations of the processes underlying body ownership. The original and most studied of these illusions is the rubber hand illusion. Synchronously touching a hidden real hand with touches delivered to a fake (rubber) hand elicits feelings of ownership over the fake hand, whereas asynchronous touch significantly reduces this effect^1^.

The rubber hand illusion has since been expanded to incorporate other body parts and even the whole body^2^, for which synchronous visuo-tactile stimulation induces feelings of ownership over an entire mannequin body viewed from a first-person perspective^2^,^3^. Such illusions are demonstrated by subjective reports of ownership as well as objective measures. For example, attacking the ‘owned’ body (part) with a threatening object can cause heightened skin conductance response (SCR) as if the real body were threatened^4^,^5^. Modulation of these illusions can alter participants’ perceptual experiences of the size and shape of their actual body (e.g. longer arms^6^ or fingers^7^, and smaller or larger bodies/body parts^8^,^9^,^10^). As well as asynchrony of touch reducing ownership, illusion strength is also affected by other factors such as a humanoid shape of the body or body part^2^,^11^, distance of the rubber hand from the body, for which peripersonal space seems to be a critical constraint^12^,^13^,^14^,^15^, and visual perspective^16^,^17^,^18^. Indeed, placing a hand at 180° from the body so that it is no longer in ego-centric coordinates (i.e., no longer viewed from a first-person perspective) is frequently used as an additional control condition^16^,^19^.

Some studies, however, claim that it is possible to elicit multisensory body illusions when the body is viewed from a third-person (observer) perspective far from the body, i.e., outside peripersonal space^20^,^21^,^22^,^23^,^24^. This standpoint asserts that the visual perspective (first or third-person) and the distance to the seen body (within or outside peripersonal space) is not important for the full-body illusion and that only visuo-tactile synchrony and humanoid shape matter^25^,^26^. However, this view disregards peripersonal space constraints of body illusions and the nature of ego-centric spatial reference frames (body-, and body-part-centred coordinates)^12^,^15^,^16^,^17^,^27^. Thus, these third-person perspective illusions may rely more on self-recognition than body ownership mechanisms, in that the participant recognises the observed body as a virtual *representation* of their own body instead of experiencing a genuine body illusion such as the rubber hand illusion^2^,^28^. This self-recognition is facilitated by immersing participants in a virtual environment, which is essential for full-body illusion paradigms in which participants are required to wear a virtual reality headset^18^,^25^.

According to this approach, when observing a body from the third-person perspective that is several metres in front of your own body, you are not directly projecting ownership and tactile sensations to this body (as in the rubber hand illusion); instead, the experience is more akin to recognising yourself in a photograph. To test this idea, Petkova and colleagues^18^ directly compared the full-body illusion elicited over a mannequin body from a first-person perspective (within peripersonal space), with that when the mannequin was viewed from the third-person perspective (outside peripersonal space), and only found evidence of a genuine body illusion using a first-person perspective. Although for non-mirror third person perspective there was no evidence for an illusion through objective skin conductance responses, ownership was not denied for this condition in the questionnaire; instead the participants were on average uncertain. This putative weak subjective “illusion” was attributed to partial self-recognition, interpreting the mannequin as representing a video image of themselves. More recent studies that also employed virtual reality technology demonstrate significantly stronger illusions for first- compared to the third-person perspective^29^ or fail to replicate key elements of the illusion from the third-person perspective^30^. Therefore, although the literature is mixed with regard to the importance of visual perspective for full-body illusions, there is growing evidence that these types of illusions are constrained by the same rules that govern the rubber hand illusion^27^.

In everyday life, the most frequent way in which we view our own body from an observer perspective is using mirrors. Few animals aside from humans can recognise themselves in a mirror, and this ability has even been proposed as a measure of self-consciousness^31^. However, does mirror self-recognition utilise the same mechanisms as recognising oneself in a photograph or a virtual representation of our body in a computer game or experimental setup^25^? Humans use mirrors not only to recognise themselves but also for visual guidance of complex actions, such as shaving or driving a car. These tasks are achieved because our brains learned the mirror transformation in early infancy^32^; thus, we intuitively understand how mirror reflections are left-right reversed and relate to our real bodies located in front of the mirror^33^. This understanding suggests that viewing the self in a mirror involves a spatial transformation process over and above pure recognition of oneself in pictures and images.

Recent neuropsychological evidence also implies a special role of mirror view for body ownership. In somatoparaphrenic patients, the sense of ownership over a disowned hemiplegic limb can be temporarily restored during mirror self-observation^34^; when the patients viewed their own hand directly, they attributed ownership of the limb to someone else, whereas viewing the limb through a mirror reflection, enabled them to claim it as their own. These findings may reflect an independent route by which body representations are updated through mirrors. However, the reinstatement of ownership reversed when the patients were no longer looking in the mirror, which may be more compatible with self-recognition mechanisms. The patients recognise that the arm is theirs when they see it as part of the self that they recognise in the mirror, but this does not actually modulate body ownership; therefore, when the mirror is removed, the arm is still disowned.

Limb ownership in a mirror has also been assessed in healthy controls using the rubber hand illusion. Bertamini and colleagues found that synchronous visuo-tactile stimulation could successfully elicit illusory ownership over a rubber hand that was viewed only through a mirror. Illusion strength was assessed via subjective reports and proprioceptive drift (re-localisation of perceived limb position towards the fake hand)^35^. Interestingly, replacing the mirror with another person’s hand or a fake hand at the corresponding location as the mirror reflection eliminated the illusion; thus, the mirror itself was critical for successful induction of the illusion when the limb was not viewed from a first-person perspective. However, it is unclear to what extent it is possible to conclusively differentiate between body ownership and self-recognition in these experiments because the authors did not directly compare mirror and non-mirror conditions. While a recent study that directly compared conditions of mirror and non-mirror viewing of a moving rubber hand replicated Bertamini’s results^36^, another study found evidence of an illusion even when the hand that was viewed in the mirror was incongruent with a reflection of the actual body. The experience of the illusion in this body-incongruent mirror condition was equivalent in strength to that in a body-congruent mirror condition, and illusion strength in both mirror conditions was significantly reduced compared to direct (first-person) viewing of the rubber hand^37^. Thus, although Bertamini’s results make sense in that visual information derived from the mirror reflection of the hand could contribute to the rubber hand illusion, the relationship between mirror reflections and limb ownership is still not fully understood.

Mirrors have also been incorporated into full-body illusions; however, in the majority of studies, the mirror was not the main mode of viewing the body^38^,^39^,^40^. In such experiments, when the participants look down, they see a computer-generated avatar’s body from a natural first-person perspective in peripersonal space. However, when they look up they see a tall mirror at some distance in which they can see a reflection of the avatar’s body and face. Thus, because the illusory ownership was triggered by a first-person perspective of the body, any contributions of the mirror reflection to the illusion experience are unclear. One study, however, has used a mirror view of a full avatar body for the illusion without also incorporating first-person perspective. In their study, Gonzalez *et al.*^41^ examined illusory ownership over an avatar viewed in a mirror using synchronous and asynchronous actions. The role of action in body illusions is debated^19^,^36^,^42^,^43^,^44^,^45^, and voluntary actions recruit additional processes such as a sense of agency. Moreover, because the specific effect of the mirror transformation was not investigated directly, no comparisons were made to non-mirror conditions.

The current experiment investigated the full-body illusion over a passive mannequin body viewed in a mirror. Over a series of experiments, both subjective and objective measures were obtained following synchronous (illusion) and asynchronous (control) visuo-tactile stimulation. The strength of the illusions via mirror view was directly compared to conditions in which the mannequin body was viewed from a non-mirror third-person perspective (see Fig. 1) as well as from a first-person perspective. We hypothesised that viewing a synchronously touched mannequin body through a mirror would elicit illusory ownership over the mannequin that would be stronger than that elicited when the mannequin was viewed from a non-mirror third-person perspective (Experiment One) and equivalent to that elicited from a first-person perspective (Experiment Three). We also predicted that synchronous touch would increase skin conductance response to a knife threat (an objective measure of body ownership) when the mannequin was viewed through the mirror but not when the mannequin was viewed from a non-mirror third-person perspective (Experiment Two).

## Results

### Experiment One: mirror vs. non-mirror, questionnaire data

#### Illusion scores

Twenty-four healthy participants took part in Experiment One, in which the subjective strength of the full-body illusion elicited when a mannequin body was viewed in a mirror was compared to that elicited when a mannequin body was viewed from a non-mirror third-person perspective. A composite illusion score^35^,^36^,^37^ was calculated for each condition by averaging across all three illusion-related questions (for a breakdown of the median values and interquartile ranges for each question, see Table 1).

The data were ordinal and not normally distributed (Shapiro-Wilk test), so were analysed using non-parametric Wilcoxon signed-rank tests. To directly test for the effect of synchrony, planned comparisons were conducted between the synchronous and asynchronous illusion scores. Synchronous trials resulted in stronger agreement to the illusion-related questions for both the mirror (z = −3.9, p < .001; synchronous median = 1.17, asynchronous median = −1.0) and non-mirror third-person perspective (z = −4.08, p < 0.001; synchronous median = 0.67, asynchronous median = −1.67) conditions (see Fig. 2).

To test the hypothesis that the mirror conditions would result in a significantly stronger illusion than the non-mirror conditions, a planned comparison was conducted between the illusion scores for the synchronous trials in the mirror vs. non-mirror conditions. Significantly greater illusion scores were found in the mirror compared to non-mirror conditions (z = −2.3, p = 0.02). Moreover, although the median values for both conditions were greater than zero, only the median for the mirror condition was above +1. An average group score of ≥+1 has been identified as a critical threshold that affirms the illusion on a group level^19^. Thus, whereas the illusion was largely affirmed in the mirror condition (median = 1.17), in the non-mirror condition, it was not (median = 0.67).

#### Control scores

Items were also included in the ownership questionnaire to control for suggestibility. These statements should not contribute to the illusory experience and thus should not be affirmed by participants. To evaluate this, control scores were calculated by averaging responses to the two control questions across the synchronous conditions, and these scores were then compared to the equivalent illusion scores using Wilcoxon signed-rank tests. As predicted, the illusion scores were significantly greater than the control scores for both mirror (z = −3.15, p = 0.002; illusion median = 1.17, control median = −0.25) and non-mirror (z = −3.03, p = 0.002; illusion median = 0.67, control median = −1.0) conditions.

### Experiment Two: mirror vs. non-mirror, SCR data

Fifty healthy participants took part in Experiment Two, in which the objective strength of the full-body illusion over a mannequin body viewed in a mirror (skin conductance response to the mannequin being threatened) was compared to responses when the mannequin body was viewed via a non-mirror third-person perspective. The SCR data were not normally distributed (Shapiro-Wilk test), so were analysed using non-parametric Wilcoxon signed-rank tests. To determine the effect of synchronous touch on SCR, planned comparisons were conducted between synchronous and asynchronous trials for both the mirror and non-mirror conditions. In the mirror condition, significantly greater SCRs were recorded following synchronous (median = 0.193 μmho) compared to asynchronous (median = 0.087 μmho) touch (z = −2.11, p = 0.035). There were no significant differences between synchronous and asynchronous for the non-mirror conditions (z = −0.17, p = 0.86). However, direct comparisons of SCR elicited by synchronous touch in the mirror vs. non-mirror conditions revealed no significant difference (z = −0.57, p = 0.57) (see Fig. 3).

### Experiment Three: mirror vs. first-person perspective, questionnaire data.

#### Illusion scores

Twenty healthy participants took part in Experiment Three, in which the subjective strength of the full-body illusion over a mannequin body viewed in a mirror was compared to that elicited when the mannequin body was viewed via a first-person visual perspective. The questionnaire results for Experiment Three were collated and analysed in the same way as for Experiment One. An illusion score was calculated from the average of the responses to the illusion-relevant questions for each condition (for a breakdown of the median values and interquartile ranges for each question, see Table 1). The data were ordinal and not normally distributed (Shapiro-Wilk test), so were analysed using non-parametric Wilcoxon signed-rank tests. To determine the effectiveness of the illusion, planned comparisons were conducted between synchronous and asynchronous illusion scores for both the mirror and first-person perspective conditions. Synchronous trials resulted in higher illusion scores compared to asynchronous trials in both the mirror (z = −3.52, p < 0.001; synchronous median = 1.33, asynchronous median = −0.67) and first-person perspective (z = −3.6, p < 0.001; synchronous median = 1.67, asynchronous median = −0.25) conditions (see Fig. 4).

To test the hypothesis that the mirror and first-person perspective conditions would result in equivalently strong illusions, a planned comparison was conducted between the synchronous illusion scores for the mirror vs. first-person perspective conditions. No significant difference was found (z = −.87, p = .383). Additionally, the median response for both conditions was greater than +1 (mirror = 1.33; 1pp = 1.67), demonstrating that in both mirror and first-person perspectives, the illusion was affirmed on the group level.

#### Control scores

As in Experiment One, control scores were calculated by averaging the responses to the two control questions in the synchronous conditions. These scores do not reflect the illusory experience; instead, they are included to control for suggestibility (see above). As predicted, the levels of agreement for the control scores was significantly lower than those for the illusion scores, in both the mirror (z = −3.92, p < .001; illusion median = 1.33, control median = −1.25) and first-person perspective (z = −3.57, p < .001; illusion median = 1.67, control median = −0.25) conditions.

## Discussion

The current study investigated whether viewing a mannequin body in a mirror modulated the strength of the full-body illusion. Touching a participant’s unseen real body in synchrony with touch delivered to an observed mannequin body elicited feelings of ownership over the mannequin when it was viewed in a mirror that was positioned as if directly in front of the participant. The illusion induced using the mirror was as strong as that induced when the mannequin was viewed from a first-person perspective and was greater than that when the mannequin was viewed from a third-person perspective without a mirror. These differences were revealed not only by the significant differences between the conditions, but also because at a group level the participants affirmed the illusion in both the mirror and first-person conditions (median value of greater than +1) but not in the non-mirror condition. Finally, the SCR experiment provided objective physiological evidence that a genuine body illusion was elicited in the synchronous mirror condition, which is in accordance with the results of earlier studies in which a body was viewed from the first-person perspective^2^,^9^. Collectively, these results support previous findings suggesting that visual perspective is a key factor for the full-body illusion^18^. They also highlight the importance of mirrors for body perception, suggesting that viewing oneself in a mirror can directly affect perceptual body representations.

As described in the introduction, several studies claim that the rubber hand illusion can be elicited when the rubber hand is viewed through a mirror, although it remained unclear whether such an illusion reflected body ownership or self-recognition^35^,^36^,^37^. When one sees a hand reflected in a mirror, it is not usually in isolation; the mirror also reflects other parts of the body that may aid recognition, such as the face. Although Bertamini and colleagues^35^ also examined this aspect and found that obscuring the face in the mirror during the mirror rubber hand illusion did not eliminate the illusion, the participants could still see other parts of their actual body, and the face-obscured condition was not directly compared to conditions in which the face was visible. In the current full-body paradigm, however, none of the participant’s actual body was in view to aid recognition. Moreover, although the participants were wearing a virtual reality headset, the observed differences in both subjective illusion strength and objective threat responses relative to these measures in the non-mirror third-person view suggest that viewing the body in the mirror can directly tap into the central body representation and affect body ownership mechanisms.

How do mirror reflections influence central body representations? We argue that visual information from the mirror reflection of the body is referred back to the body in front of the mirror. Therefore, just as we do not reach towards the images of objects that we see reflected in a mirror, during the mirror full-body illusion we do not experience a referral of touch towards the location of the observed image or feel as though we are occupying the same spatial position as the image that we see. Instead, due to our inherent knowledge of mirror transformations, which is learned in very early childhood, visual information from the mirror reflection is translated back to our own body and can influence multisensory body representations.

We know that the mirror conditions of the current experiment created the experience of looking in a mirror, as demonstrated by the participants’ agreement with the questionnaire statement “*it felt like I was looking in a mirror*” in the mirror synchronous conditions for both experiments one and three (see Table 1). For all other conditions, including the mirror asynchronous conditions, the median responses to this question were below the critical value of +1 that reflects affirmation of the given perceptual experience, which suggests that, at the group level, the participants did not feel as though they were looking in a mirror. We therefore suggest that the experience of looking in a mirror and seeing the mannequin body being stroked synchronously with the touches felt on the participants’ own unseen bodies made the participants experience ownership of a mannequin standing directly in front of the mirror. Thus, the visual information from the reflection was referred back to the peripersonal space around the participant’s own body, and this visual information was then combined with tactile and proprioceptive information according to an ego-centric spatial reference frame centred on the unseen actual body (in front of the mirror). This interpretation is in accordance with behavioural data from cross-modal congruency tasks that have shown that visual reflections in mirrors can lead to visuo-tactile interactions that are specific to peripersonal space and similar to those observed when the hand is viewed under natural viewing conditions without mirrors^46^.

By contrast, in the non-mirrored third-person perspective condition, the absence of the mirror means that the spatial mirror transformation is not engaged; as a result, the participants experience looking directly at a mannequin that is one and a half metres in front of themselves and not a reflection of a mannequin standing in peripersonal space. Therefore, the illusion that is elicited in the non-mirror condition is significantly weaker because the visual information is attributed to events that occur outside of the peripersonal space of the participant. This spatial discrepancy obstructs ego-centric integration of visual, tactile, and proprioceptive signals into a coherent multisensory percept of a single body, just as placing the fake hand far from the body reduces ownership in the traditional rubber hand illusion^7^,^12^,^13^. Instead, participants experienced the touches that they saw applied to the mannequin and the touches that they felt on their own body as two spatially distinct events, eliminating (or significantly diminishing) illusory feelings of ownership over the mannequin body. This reduction in illusion strength during the non-mirror condition supports the assertions of Petkova *et al.*^18^, who stated that visual perspective is imperative for the full-body illusion and that visuo-tactile synchrony and a humanoid shape are not sufficient to elicit strong feelings of ownership over a fake body^25^. Indeed, the results of the current study suggest that although viewing our own bodies in a mirror enables us to see ourselves from a different angle, akin to how an observer would view us (third-person perspective), we never actually feel as if we are standing in the mirror (the location of the observed image). Instead, our knowledge of the mirror transformation means that we relate every image we see reflected in the mirror back to the world directly in front of the mirror, including our own body. Therefore, a mirror view of one’s own body is equivalent to a first-person view of one’s own body in terms of multisensory integration in peripersonal space.

With regard to clinical implications, the current findings cannot answer the question of whether restored limb ownership in somparoparaphenria patients looking in a mirror^34^ was due to self-recognition or body ownership. Future experiments should specifically examine self-recognition and its effect on restored ownership in somatoparaphrenia (for example, mirror view with and without the face) as well as brain activity during mirror and direct views of one’s own body to identify differences and similarities in the underlying neural circuitry. Furthermore, because a mirror view allows a third-person view of one’s own body, it may be more important for affective body representations (how we feel about our body). First-person modulation of perceived body size using multisensory illusions has already been found to directly affect body satisfaction^9^. Because of the social implications of mirror view (seeing your own body as others see you), body size modulations while looking in a mirror may have greater effects on our emotions.

The skin conductance data revealed a significant effect of synchrony in the mirror condition but not the non-mirror condition. These data are important because they provide objective physiological evidence that a genuine body ownership illusion was elicited in the mirror condition, in accordance with earlier studies that have used this measure for the rubber hand illusion^4^,^5^ and the first-person perspective full-body illusion^2^,^9^. Thus, we have complementary subjective and objective evidence in support of our main conclusion that the full-body ownership illusion can be induced in a mirror. However, subsequent analysis that directly compared responses in the synchronous trials demonstrated no significant difference between mirror and non-mirror conditions. In the non-mirror third-person condition, the mannequin body was positioned in place of the mirror and consequently appeared larger than the mirror reflection of the mannequin. This apparent size difference occurred because the distance between the mannequin and the mirror was also reflected, such that the mannequin in the mirror appeared farther away and hence smaller. As a consequence, not only was the image of the mannequin slightly larger in the non-mirror condition but the image of the knife during the knife threat was also larger for the same reason. This seemingly larger knife could have resulted in higher SCR during the non-mirror conditions regardless of ownership, which would explain the lack of a significant difference between the synchronous trials of the mirror and non-mirror conditions. This difference between conditions highlights the importance of the asynchronous control trials. The asynchronous trials utilized identical visual and tactile input to that in the synchronous trials (the same video and number of touches) but with vision and touch uncoupled by the delay applied to the audio cues and hence the felt touch therefore allowing statistical comparison of otherwise identical conditions.

The overall experience of an illusion, such as the full-body illusion, is thought to comprise of different components. These components include referral of touch, - the feeling that the touch one feels are the same as those one sees (touching the mannequin), as well as explicit feelings of ownership - when the mannequin body feels like one’s own body, both of which were addressed by items in the current ownership questionnaire. The majority of past studies, including those examining feelings of limb ownership in a mirror^35^,^36^,^37^, focused on the overall illusion experience and not the constituent parts. Therefore, our predictions did not include differences between these subcomponents. Nevertheless, retrospective examination of the median scores for the individual questionnaire items may suggest that the item directly pertaining to body ownership – *“It felt like the mannequin’s body was my body”* – was most affected by visual perspective, whereas on average, participants still expressed some level of agreement with the referral of touch items in the non-mirror third-person perspective condition (see Table 1). Thus, weak residual illusions observed in third-person perspective conditions in this and in previous experiments might be driven by specific subcomponents of the illusion. Thus, it may be that visuo-tactile synchrony is sufficient to elicit some degree of referral of touch sensations, but adherence to constraints of peripersonal space (via direct view or through a mirror) is essential for a full sense of body ownership. However, the current experiment was not designed to tease apart these different aspects of the illusory experience. Future studies that aim to examine the subcomponents of the full-body illusion (both for the basic illusion and for different manipulations such as mirror/non-mirror views) should include more detailed questionnaire items similar to those employed for the rubber hand illusion^47^ and a larger sample size to account for the analyses required to tackle these questions.

In sum, the current findings support the notion that multisensory body illusions that are elicited as if looking in a mirror update human central body representations in a similar way to normal first-person view of the body. Touches to one’s own body that are synchronous with those applied to a mannequin viewed in a mirror induce strong feelings of ownership over the mannequin body, which is experienced as if standing in front of the mirror in peripersonal space. This effect of mirror viewing is distinct from conditions in which the mannequin is viewed from a non-mirror third-person perspective, when much weaker illusion scores are observed and physiological responses to threats against the mannequin are not affected. These findings are compatible with the assertion that visual perspective plays an important role in body ownership and also suggest that mirrors are special for viewing the self by providing a unique first-person perspective of our body from the outside.

## Methods

### Experiment One

#### Participants

Twenty-four participants (13 female and 11 male) with a mean age of 27 years (range = 19–42) took part in the first experiment. All participants had normal or corrected-to-normal vision, no history of psychiatric disorders (self-reported), and gave written informed consent. All experiments were conducted in accordance with the Declaration of Helsinki and were approved by the Swedish Central Ethical Review Board.

#### Materials and methods

During the experiment, the participants wore a set of head-mounted displays (HMDs) (VR1280; Virtual Research Systems, Inc, Aptos, California, USA) with a 60° field of view and a display resolution of 1280 × 1024. One male and one female shop mannequin were used to make the videos and as props during the experiment. A stick (92 cm in length) with a polystyrene sphere on the end (6 cm in diameter) was used to deliver the touches. The ownership questionnaire that was used to quantify the subjective strength of the ownership illusion was based on a previous study^18^ and was delivered via the HMDs.

##### Pre-recorded videos

**Figure 1:**
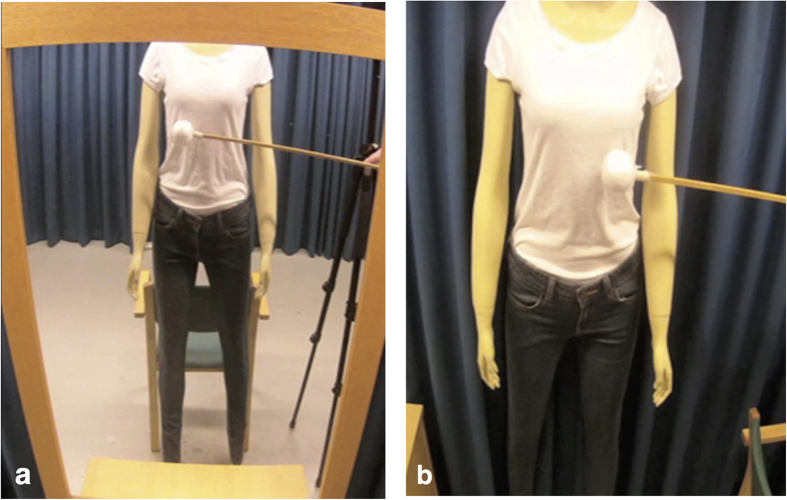
Stills from the videos of the female mannequin in the mirror (**a**) and non-mirror (**b**) third-person perspective conditions.

**Figure 2:**
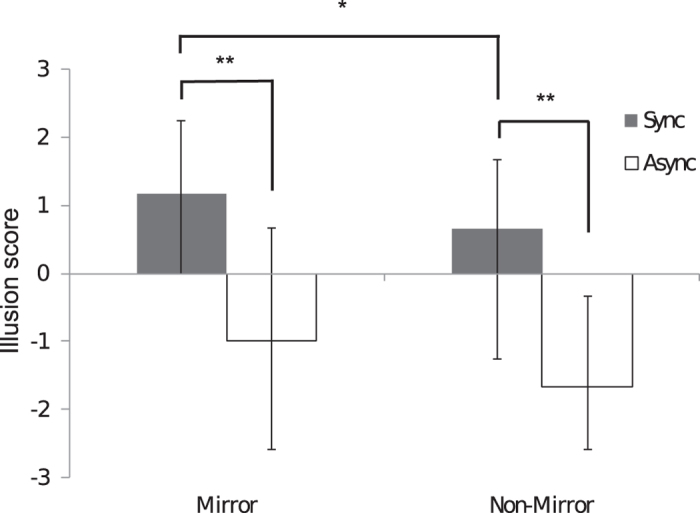
Synchronous touch produced a significantly stronger illusion in the mirror compared to the non-mirror third-person perspective condition. Higher illusion scores were found following synchronous (closed bars) compared to asynchronous (open bars) touch for both the mirror and non-mirror conditions. The graph depicts the median values, and the error bars represent the interquartile range.

**Figure 3:**
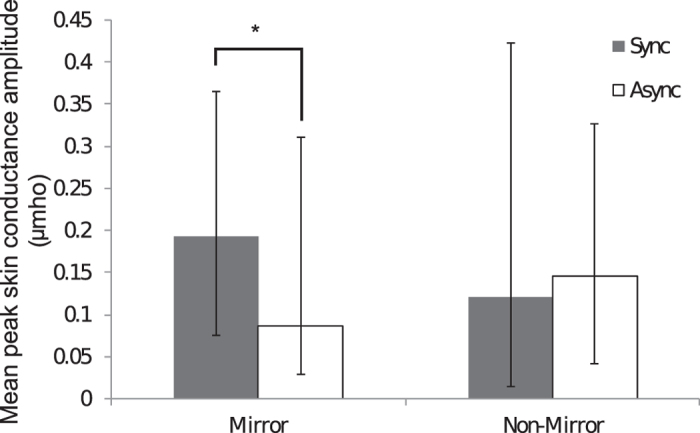
The amplitude of the skin conductance response elicited by the knife threat was significantly greater following synchronous (closed bars) compared to asynchronous (open bars) touch in the mirror condition. Synchrony did not have a significant effect in the non-mirror third-person perspective condition. The graph depicts median values, and the error bars represent interquartile range.

**Figure 4:**
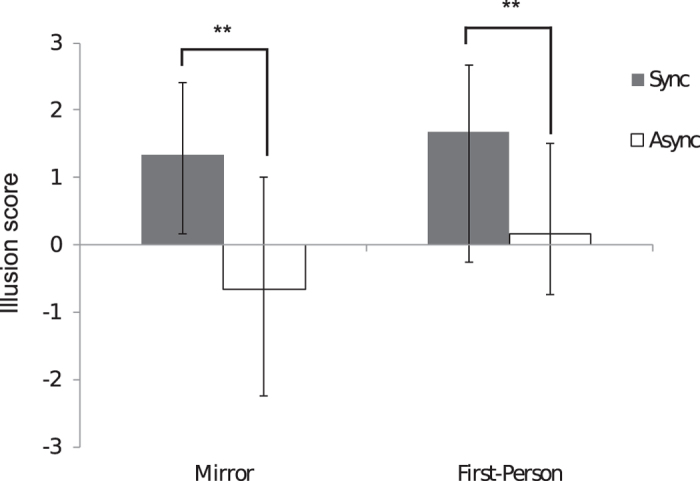
Illusion scores were significantly greater following synchronous (closed bars) compared to asynchronous (open bars) touch for both the mirror and first-person perspective conditions. Visual perspective (mirror vs. first person) did not have a significant effect when the touch was synchronous. The graph depicts median values, and the error bars represent interquartile range.

To present images of the mannequins in the different conditions (see Fig. 1), videos were pre-recorded. In these videos, the mannequin bodies were either reflected in a mirror (from the perspective of the mannequin head) or viewed directly (see the supplementary videos). For the mirror videos, a mirror (75 cm × 100 cm inclusive of its frame, with a reflective surface of 62 cm × 87 cm) was leaning against the wall on top of a cabinet. The mannequin was positioned approximately 1.5 metres in front of the mirror. A standard camera tripod was placed to the right (mannequin perspective) of the mannequin. A custom-made weighted metal mount was positioned on top of the tripod. This mount projected 15 cm leftwards from the top of the tripod and held two identical cameras (CamOne Infinity HD, resolution 1920 × 1080, Touratech AG, Germany) directly above the mannequin (at the position of the eyes). The angles of the cameras and mirrors were positioned such that the recorded images captured the mirror reflection of the mannequin body from the top of the shoulders to the ankles.

A chair was placed directly behind the mannequin and kept in place during the experiments. This chair helped stabilise the mannequin in a standing position whilst the videos were recorded. The chair also indicated where the participant should stand (directly in front of the chair), helped the participant to remain stable throughout the experiment, and acted as a mirror cue (the participants stood in front of a chair and then saw that same chair in the mirror behind the mannequin). The tripod could also be seen in the mirror images and therefore was also left in place during the experiments to act as a mirror cue. The experimenter then used a stick to stroke the torso of the mannequin body in time with a pre-recorded audio cue that was played back to the experimenter through headphones during the experiment. An equal number of touches were delivered to the left, right, and centre of the torso. Each touch lasted for approximately one second, starting at the bottom of the rib cage and moved downward, finishing just below the waist. Because the cameras were positioned directly above the mannequin, care was taken during filming of the videos to ensure that no part of the mannequin or the stick could be observed from a first-person perspective and that both the mannequin and stick could only be observed in the mirror. Part of the cabinet could also be seen in the lower portion of the mirror, which obscured view of the mannequin’s feet. In the non-mirror videos, the mirror and cabinet were moved out of view and replaced with the mannequin. Therefore, the cameras captured the mannequin body from the top of the shoulders to the ankles, similar to the images that were captured during the mirror condition. However, the crucial difference was that in this condition, the participants seemed to look directly at the mannequin body as opposed to seeing a mirror reflection of the mannequin as described above.

In all conditions, stroking lasted approximately 2 minutes from the beginning of the first to the end of the last touch. Additionally, for all conditions, the touches that were applied to the participant corresponded to those applied to the mannequin, i.e., a touch to the right side of the participant’s torso was matched by a touch to the right side of the mannequin’s torso. Due to the left-right reversal of mirror reflections, touching the right side of the mannequin torso visually appears like touching the left side of the mannequin. Separate videos were made for male and female participants using sex-matched mannequins. The mannequins were dressed in blue jeans and white t-shirts and had the head removed at the neckline to enable correct positioning of the cameras. In all of the experiments, the participants were asked to wear a similar outfit, and the majority of them complied.

#### Procedure

The experiment utilised a within-participants design. The participants wore the HMDs and stood facing the mannequin and the mirror with their heads facing forwards, as if they were looking straight ahead. Each pre-recorded video began with 5 seconds of blank screen followed by 2 minutes of touches. During the touches, the mannequin was viewed in the mirror or from a third-person perspective without the mirror using the appropriate video. For each trial, the experimenter stroked the participants’ actual torso in time to the same audio cues used to make the video. These cues were played through headphones, so they were not audible to the participants. In the synchronous conditions, the timing of the cues played to the experimenter, and thus the touches delivered to the participant, was identical to video. In the asynchronous trials, a delay of 1000 ms was applied to the audio cues so that the touches that were observed in the video and the touches that were felt were delivered at different times. The touches were delivered in an unpredictable pattern, although they were divided equally between the centre, left, and right side of the torso. A total of 60 touches were applied in each condition. Following each condition, the questionnaire items were presented in a random order through the HMDs. The participants were required to verbally respond, and the experimenter recorded these responses. Each participant took part in all of the conditions (mirror and non-mirror with both synchronous and asynchronous touch), the order of which was counterbalanced between participants.

### Experiment Two

#### Participants

A new cohort of fifty participants (23 male and 27 female) with a mean age of 26 years (range = 18–56) took part in Experiment Two. As in Experiment One, all participants had normal or corrected-to-normal vision, no history of psychiatric disorders (self-reported) and gave written informed consent prior to taking part in the study.

#### Materials and Methods

The materials and methods were the same as those for Experiment One except that the questionnaire was not used and skin conductance response (SCR) were recorded using a pre-established protocol^2^,^9^. The SCR measurements were taken using a Biopac System MP150 (Goleta, USA), with the gain switch set to 5 mmho/V and a CAL2 Scale Value of 5. Electrodes were attached to the index and middle fingers of the participant’s left hand using Signa electrode gel (Parker Laboratories, INC., New Jersey, USA). Skin conductance, as a measure of autonomic arousal, was recorded at 100 samples per second and processed with the Biopac software package Acknowledge for Windows (ACK100W).

Separate pre-recorded videos were created for Experiment Two. These videos were identical to those used in Experiment One, with the addition of a knife threat and the placement of skin conduction sensors on the mannequin’s fingers. The knife threat was achieved using a 33 cm kitchen knife (21 cm blade), which approached the mannequin body from the *right* side (participant perspective) in a stabbing motion (see supplementary videos). The SCR was defined as the peak amplitude that occurred within five seconds after the onset of the knife threat and was calculated by identifying the maximum conductance value and then subtracting the minimum preceding value within the 5-second time frame. In each trial, the experimenter initiated recording of the SCR data by pressing a key when the knife entered the image. The knife was in the image for approximately two seconds. All participants were included in the analysis; therefore, trials without any response (and null responders)^48^ were included in the calculation of SCR magnitude^49^. Different experimenters acquired and analysed the data, and the data were extracted using an automated computer program.

#### Procedure

The procedure for Experiment Two was identical to that of Experiment One, except that the touches were applied for approximately 1 minute per trial (from the beginning of the first touch to the end of the last touch). In addition, immediately following the touches, the participants observed the mannequin being threatened with a knife (see above). instead of responding to the questionnaire. In accordance with previous studies^2^,^8^, the skin conductance measures were acquired over a short time period because cognitive-based subjective responses are believed to require greater immersion in the experiment and hence a longer duration of acquisition compared to more automatic physiological responses such as SCR. For this experiment, each participant took part in three trials for each condition; the order of these trials was counterbalanced across participants.

### Experiment Three

#### Participants

A new sample of twenty participants (10 male and 10 female) with a mean age of 28 years (range = 18–56) took part in the third experiment. All participants had normal or corrected-to-normal vision, no history of psychiatric disorders (self-reported), and gave written informed consent prior to taking part in the study.

#### Materials and methods

The materials and methods were the same as those used as in Experiment One, except that new videos were created in which the mannequin bodies were viewed from a first-person perspective. During filming, the cameras were positioned at the level of the mannequin’s eyes facing downward to capture the image of the body from above.

#### Procedure

The procedure for Experiment Three was identical to that used in Experiment One, but this time, the mirror view was compared with the traditional first-person perspective view of the mannequin body. The participant’s body was touched with the stick in time to the audio cues that were presented to the experimenter (not the participant) either synchronously or asynchronously with the touches that were delivered to the mannequin (on the pre-recorded video). After each trial, which lasted approximately 2 minutes, the perceived strength of the body illusion was quantified using the same questionnaire that was used in Experiment One.

## Additional Information

**How to cite this article**: Preston, C. *et al.* Owning the body in the mirror: The effect of visual perspective and mirror view on the full-body illusion. *Sci. Rep.* **5**, 18345; doi: 10.1038/srep18345 (2015).

## Supplementary Material

Supplementary Video

Legend for Supplementary Video

## Figures and Tables

**Table 1:** Median values and interquartile ranges (IQRs) for each question in experiments one and three.

Question	Experiment One: Median (IQR)				Experiment Three: Median (IQR)			
	Mirror sync	Mirror async	Non-mirror sync	Non-mirror async	Mirror sync	Mirror async	1pp† sync	1pp† async
I seemed to feel the touch given to the mannequin	2 (0.25–3)	−.5 (−2.75–1.75)	1.5 (−0.75–2)	−2 (−3–1.75)	2 (1.25–3)	−0.5 (−3–1)	2 (1.25–3)	0.5 (−2–2)
It seemed as though the touch I felt was caused by the stick touching the mannequin’s body	1 (−1–2)	−1 (−3–1)	1 (−1–2)	−2 (−2.75–0)	1.5 (1–3)	0 (−2–1.75)	2 (1–3)	0 (−2–1.75)
It felt like the mannequin’s body was my body	1.5 (−1–2)	−2 (−3–−1)	−0.5 (−2–1.75)	−3 (−3–−2)	2 (0.25–2)	−2 (−3–0.75)	2 (1–2.75)	0.5 (−2–2.75)
I felt like I had two bodes (at the same time)	0.5 (−1–2)	−2 (−3–1)	0 (−2–1)	−2 (−3–−1)	−2 (−3–0)	−2 (−3–1)	−0.5 (−2.75–1)	−1.5 (−3–1)
It felt as if my body had turned into a plastic body	−0.5 (−2–1)	−1.5 (−3–0)	−1 (−2.75–0)	−3 (−3–−2)	−1 (−3–1)	−2 (−3–0.75)	−0.5 (−3–2)	−2 (−3–1)
I felt like I was looking in a mirror	1.5 (0–3)	0.5 (−2.75–1)	0 (−2–2)	−2 (−3–−1.25)	2 (0–3)	−0.5 (−2–1.75)	−2 (−3–1)	−2 (−3–−1.25)
It felt as if the body I saw belonged to someone else	−1 (−1.75–1)	2 (1–3)	1.5 (0–2)	2 (2–3)	−1 (−2–1.75)	2 (−0.75–2.75)	−1.5 (−2–1)	1 (−2–2)

^†^1pp = first-person perspective.
