# Supplementary material for: Owning the body in the mirror: The effect of visual perspective and mirror view on the full-body illusion
Source: Sci Rep. 2015 Dec 17;5:18345. doi: 10.1038/srep18345 (PMC4683587; doi:10.1038/srep18345)
Supplement: Legend for Supplementary Video [file srep18345-s2.pdf]

Owning the body in the mirror: The effect of visual perspective and mirror view on the full-body illusion

Authors: Catherine Preston[1], Benjamin J Kuper-Smith[1], and H. Henrik Ehrsson[1]

Affiliations: [1]Brain, Body and Self Laboratory, Department of Neuroscience, Karolinska Institutet, Stockholm, Sweden

Legend for supplementary video

Supplementary Video: Video images demonstrating all the different trial types (synchronous and asynchronous with third-person, mirror third-person, and first-person perspectives) for both male and female participants. Both the participant (left part of the screen) and participant view (right part of the screen) are shown. The video also demonstrates the visually presented knife threats.
